# In Silico Evaluation of In Vivo Degradation Kinetics of Poly(Lactic Acid) Vascular Stent Devices

**DOI:** 10.3390/jfb15050135

**Published:** 2024-05-17

**Authors:** Shicheng He, Lingling Wei, Guixue Wang, Nicola M. Pugno, Qiang Chen, Zhiyong Li

**Affiliations:** 1 Biomechanics Laboratory, School of Biological Science and Medical Engineering, Southeast University, Nanjing 210096, China; 2 School of Food and Biological Engineering, Hefei University of Technology, Hefei 230601, China; 3 Key Laboratory for Biorheological Science and Technology of Ministry of Education, State and Local Joint Engineering Laboratory for Vascular Implants, Bioengineering College of Chongqing University, Chongqing 400030, China; 4 Laboratory for Bioinspired, Bionic, Nano, Meta Materials and Mechanics, University of Trento, Via Mesiano 77, 38123 Trento, Italy; 5 School of Engineering and Materials Science, Queen Mary University of London, Mile End Road, London E1 4NS, UK; 6 School of Mechanical, Medical and Process Engineering, Queensland University of Technology, Brisbane, QLD 4001, Australia; 7 Faculty of Sports Science, Ningbo University, Ningbo 315211, China

**Keywords:** biodegradable vascular stents (BVS), poly(lactic acid) (PLA), stent design, degradation model, finite element method

## Abstract

Biodegradable vascular stents (BVS) are deemed as great potential alternatives for overcoming the inherent limitations of permanent metallic stents in the treatment of coronary artery diseases. The current study aimed to comprehensively compare the mechanical behaviors of four poly(lactic acid) (PLA) BVS designs with varying geometries via numerical methods and to clarify the optimal BVS selection. Four PLA BVS (i.e., Absorb, DESolve, Igaki-Tamai, and Fantom) were first constructed. A degradation model was refined by simply including the fatigue effect induced by pulsatile blood pressures, and an explicit solver was employed to simulate the crimping and degradation behaviors of the four PLA BVS. The degradation dynamics here were characterized by four indices. The results indicated that the stent designs affected crimping and degradation behaviors. Compared to the other three stents, the DESolve stent had the greatest radial stiffness in the crimping simulation and the best diameter maintenance ability despite its faster degradation; moreover, the stent was considered to perform better according to a pilot scoring system. The current work provides a theoretical method for studying and understanding the degradation dynamics of the PLA BVS, and it could be helpful for the design of next-generation BVS.

## 1. Introduction

The permanent implantation of bare or drug-eluting metallic stents inherently limits vascular adaptability by suppressing its vasomotor responses [1,2]. Such limitations have been associated with adverse clinical outcomes, particularly restenosis and thrombosis complications [3]. Bioabsorbable and biodegradable vascular stents (BVS) were proposed as two of the five most important research topics and emerging trends in a study by Tan et al. [2], which reviewed the history, current situation, and research trends of global drug-eluting stents in the past 20 years using bibliometric methods [2]. The review indicated that BVS were promising alternatives to traditional metallic stents. Serving as temporary supports for 6–12 months, the BVS are expected to permit artery remodeling and are completely degraded within 36 months, thus reducing the risk of clinical complications [4,5,6]. One of the common materials used for fabricating BVS is poly(lactic acid) (PLA) due to its unique degradation kinetics, high biocompatibility, and good mechanical properties [7,8]. Moreover, several PLA-based vascular stents with different stent structures are currently employed in clinics. However, the in vivo degradation of these stents has not been evaluated due to complex degradation kinetics, which is challenging to characterize. Alternatively, in silico methods can quantify the degradation process by developing mathematical modeling. Therefore, studying degradation kinetics via in silico methods is very useful for understanding the entire servicing process of the BVS.

Recently, various PLA BVS were developed, and current ongoing research still endeavors to enhance the design of next-generation PLA BVS [9]. The Absorb BVS (Abbott Vascular Inc., Santa Clara, CA, USA) was the first FDA-approved stent and one of the earliest stents introduced into clinical trials [10,11,12]. The 5-year follow-up results after Absorb BVS implantation showed that the mean vascular lumen area reduced by 3.1% within 6 months and increased from 6 months to 1 year and 5 years [13]. The DESolve BVS (Elixir Medical Inc., Sunnyvale, CA, USA) has also been extensively studied clinically, with an in-stent late lumen loss reported as 0.20 ± 0.32 mm at the 6 month point [14,15]. Tamai et al. [16] conducted a 6 month follow-up study on 50 patients using the Igaki-Tamai stent (IgakiMedical Inc., Kyoto, Japan). They reported that the vessel experienced a diameter stenosis of 30% immediately after stenting, which decreased to 17% at 3 months and further reduced to 16% at 6 months. The above-mentioned stents exhibited varied clinical performances due to the different structural designs. This indicated that geometrical structure played an important role in stent degradation behaviors [17]. Although these PLA stents are evaluated by clinical trials, which stent performs better remains elusive. Thus, a comparison analysis of the degradation behaviors of PLA BVS with different structures is important to clarify the priority of the stents for the BVS selection to treat vascular diseases.

In silico methods can help understand the degradation process. As one of the methods, finite element analysis (FEA) has been widely used to design and evaluate medical devices and further optimize devices in the sense of mechanics [18,19]. Biodegradable metallic [20,21] and polymeric stents [22,23,24] were previously studied by FEA. These studies have revealed the mechanical behaviors of BVS during stent deployment, expansion and recoil, stent-balloon interaction, etc., and clearly showed that the stent’s design affects the mechanical behaviors of the stents. For example, Amineh et al. [23] developed a quasi-linear viscoelastic model coupled with a hydrolysis degradation model to explore the time-dependent mechanical behavior of PLA stents. Lounansa et al. [24] proposed a novel “circular and elliptic” cross-section of a stent bar to investigate its superior efficiency relative to other cross-section shapes under pulsatile blood pressure. However, some studies evaluated long-term effects by examining the fatigue damage of stents instead of clearly presenting the degradation dynamics [21,24]. Very recently, the authors developed a novel three-factor regulated degradation model of a stent based on a polymer degradation model and examined the stent degradation behavior within 180 days under different blood pressures [25], but some limitations still need to be overcome, such as the unreal pulsatile pressure wave and ignored fatigue effect with respect to the equivalence of the “one-cycle” to “one day”. Thus, in view of the quantifiable ability and wide application of FEA in BVS degradation, we also employ FEA here to study the BVS degradation.

Therefore, the present study aims to compare the different crimping and degradation processes of four stent structures (named Absorb, DESolve, Igaki-Tamai, and Fantom (REVA Medical Inc., San Diego, CA, USA)) from different medical companies by integrating the previous three-factor regulated degradation model and FEA. In particular, four indicators (i.e., mean number average molecular weight, residual volume fraction, mean von Mises stress, and stent diameter) were mainly collected and discussed to evaluate the four stent degradation behaviors within 180 days. Finally, a pilot scoring system was proposed to select a better stent product according to the current methods.

## 2. Methods

### 2.1. Multifactor-Regulated Degradation Model

The PLA degradation mechanism is complex and involves multiple factors, such as stress, autocatalysis, swelling, etc. Under these factors, the long molecular chains stochastically cleave into oligomers or monomers, which can be dissolved into water and thus reduce the PLA weight. Substantially, the stress reduces the activation energy of the hydrolytic reaction, the autocatalysis improves local pH values, and the swelling increases water concentrations inside the PLA. Moreover, according to the size of PLA samples, the cleavages may start at the surface or everywhere inside the polymer, and these phenomena are, respectively, called surface erosion and bulk erosion [26]. Wherein, classical pseudo first-order kinetics was widely used to illustrate the bulk erosion induced by the hydrolysis of PLA [27]. Herein, β(t) is defined as the normalized number average molecular weight, and $p\left(t\right)$ is defined as the degradation probability density of a material point due to the stochastic cleavage event:

(1) $$\left\{\begin{array}{c} \beta \left(t\right)=\frac{M_{n}(t)}{M_{n}(0)}=e^{-\lambda t}\\ p\left(t\right)=k\lambda e^{-k\lambda t} \end{array}\right.$$

where $M_{n}(t)$ and $M_{n}(0)$ represent the instantaneous and initial number average molecular weights of the PLA material points, respectively, and *k* denotes a coefficient representing the size effect of elements. Meanwhile, 𝜆 denotes the refined bulk degradation rate, which represents the same degradation kinetics of all material points at different time scales. Considering the complexity of PLA degradation, the authors previously refined the classical first-order kinetic model by introducing stress, autocatalytic, and swelling factors [25], and the degradation rate is expressed as:

(2) $$\lambda =\lambda_{0}e^{\frac{B\sigma }{RT}+C_{m}+\left(\alpha_{v}-1\right)}$$

where $\lambda_{0}$ denotes the initial degradation rate determined by the polymer components; *B* denotes the Boltzmann constant; *σ* denotes the mechanical stress acting on the PLA material point caused by the stent expansion and different blood pressures; *R* denotes the gas constant; *T* = 310 K denotes the Kelvin temperature; $C_{m}$ denotes the concentration of hydrolysates, of which the autocatalysis-induced carboxyl group is the main group; and $\alpha_{v}\;$ denotes the volume swelling factor of the PLA. The release-diffusion process of the hydrolysates is governed by Fick’s second law, and the concentration $C_{m}$ and diffusion coefficient φ are defined as follows [28]:

(3) $$\left\{\begin{array}{c} \frac{\partial C_{m}}{\partial t}=\nabla \left(\varphi \nabla C_{m}\right)+S(t)\\ \varphi ={\varphi_{0}e}^{\theta (1-\beta \left(t\right))} \end{array}\right.$$

where S(t) represents a term denoting the source of hydrolysates in a material point, $\varphi_{0}$ denotes the initial diffusion coefficient of undegraded polymer, and θ denotes a material-dependent constant. The volume swelling index $\alpha_{v}$ followed our previous derivation [25]:

(4) $$\alpha_{v}(t)=1+\frac{\rho_{dry}}{\rho_{water}}\alpha_{m}\left(t\right)$$

where $\rho_{dry}$ denotes the density of dry PLA, and $\alpha_{m}\left(t\right)$ denotes the mass swelling index. For evaluating the complete degradation of the PLA material point, we introduced two criteria: when $\beta \left(t\right)$ is below a threshold of $\beta_{thre}$ = 0.01 or the degradation probability $\int_{t}^{t+∆t}p\left(t\right)dt$ is greater than a random number *P* between 0 and 1, the PLA material point is considered to completely degrade [28]:

(5) $$\left\{\begin{array}{c} \beta \left(t\right)<\beta_{thre}\\ \int_{t}^{t+∆t}p\left(t\right)dt>P \end{array}\right.$$

The two criteria are proposed based on two considerations: (1) When the normalized number average molecular weight $\beta \left(t\right)$ of a material point degraded to a small enough value, indicating the null contribution of the material point to the stent’s mechanical behavior. (2) Probability-based judgment was introduced because PLA molecular chain cleavages are stochastic events, and it is well accepted in polymer physics.

### 2.2. Finite Element Analysis

**Figure 1:**
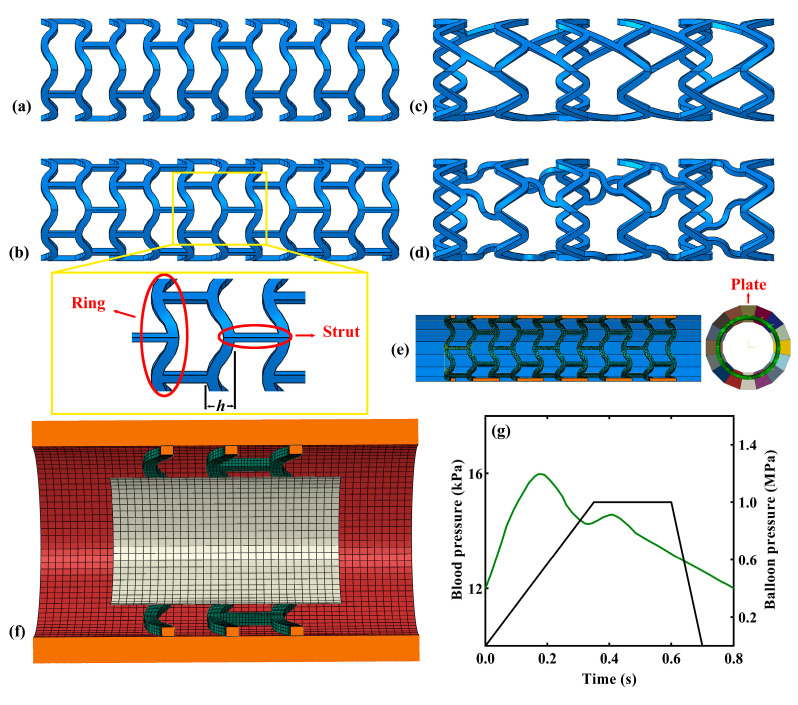
Geometrical models of the (**a**) Absorb, (**b**) DESolve, (**c**) Igaki-Tamai, and (**d**) Fantom stents; (**e**) the stent’s crimping model and plate distributions; (**f**) the stent–vessel–balloon system; (**g**) blood pressure and balloon pressure.

Geometrical model: As shown in Figure 1a–d, the names of four PLA BVS were Absorb, DESolve, Igaki-Tamai, and Fantom. Generally, a tubular vascular stent is composed of two basic units: the supporting ring and connecting strut (Figure 1b: *h* refers to the supporting ring amplitude). The Absorb and DESolve stents shared the same geometry of the supporting rings but contained different numbers of connecting struts. Similarly, the Igaki-Tamai and Fantom stents shared the same geometry of the supporting rings but had different connecting strut structures (i.e., a straight connecting strut for the Igaki-Tamai stent and a wavy strut for the Fantom stent). In detail, the geometrical parameters of the four stents were defined according to the literature [22,29] and are listed in Table 1. It is worth mentioning that the full stent model was used in the stent crimping simulation, but for the sake of saving computing cost, we selected a stent segment containing three rings in the subsequent degradation simulation without loss of generality. The vessel was modeled as an idealized straight tube (Figure 1f) with a length of 6.0 mm, an inner diameter of 3.0 mm, and a thickness of 0.4 mm. The balloon was modeled as an idealized straight tube as well (Figure 1f), for which the length, inner diameter, and thickness were 3.6 mm, 2.0 mm, and 0.06 mm, respectively. Sixteen rigid plates were uniformly distributed circumferentially around the stent to compress the stent radially in order to simulate the crimping process (refer to the sixteen pates marked with different colors in the front view in Figure 1e). Moreover, each plate was 30.0 mm in length and 0.6 mm in width.

Material model: The PLA BVS was simplified as an ideal elastic-plastic model during crimping simulation as the observed stress-strain curves of PLA at 37 °C [30], and more, the simplification, could improve computing efficiency. Herein, a Young’s Modulus of 3.0 GPa, Poisson’s ratio of 0.3, and yield strength of 50 MPa were used for the PLA [31]. It should be noted that the yield strength decreases as PLA degradation proceeds, and the decreased yield strength is expressed as follows:

(6) $$\sigma_{y}\left(t\right)=\eta \sigma_{y}\left(0\right)e^{-\Lambda t}$$

where $\sigma_{y}\left(0\right)$ denotes the initial yield strength of undegraded PLA; Λ=ϕλ denotes the decrease rate of the yield strength and was reported to be linearly correlated with the degradation rate of the PLA [32]; ϕ denotes the proportional coefficient and was calculated by parameters in the literature [32]; η denotes the strength reduction coefficient induced by PLA BVS fatigue as the stents would be subjected to cyclic blood pressure, and it was estimated by referring to the approximate fatigue limitation of 10 MPa according to the S-N curve of PLA (see the current strength decay curve of PLA in Figure S1 in Supplementary Materials) [22]. In a previous study [25], the fatigue effect was not included during the equivalence of the “one cardiac cycle” to “one day”. Moreover, the multicycle effect of the pressure during the one-day cycle would reduce the strength of the PLA. Herein, Equation (6) overcame the limitation in [25]. Simultaneously, the elastic modulus of the material also varied during the degradation process, where the complete degraded elements would be assigned with a very small modulus rather than being deleted, which was carried out in our previously published work as follows [25,28]:

(7) $$E_{s}\left(t\right)=\left(E_{s}\left(0\right)-E_{water}\right)\frac{e}{e-1}\left(1-e^{-\beta \left(t\right)}\right)+E_{water}$$

where $E_{s}\left(0\right)$ and $E_{water}$ denote the elastic moduli of the initial PLA and degradation product, which was considered as water, respectively. Equation (7) indicates that the complete degraded element is not deleted in the FE simulation but is assigned a small modulus of $E_{water}$. The vessel and balloon were considered incompressible, isotropic, and hyper-elastic materials, and this follows the Mooney–Rivlin model [33,34]:

(8) $$W=C_{10}\left({\bar{I}}_{1}-3\right)+C_{01}\left({\bar{I}}_{2}-3\right)$$

where $C_{10}$ and $C_{01}$ denote material constants; and ${\bar{I}}_{1}=\lambda_{1}^{2}+\lambda_{2}^{2}+\lambda_{3}^{2}$ and ${\bar{I}}_{2}=\lambda_{1}^{2}\lambda_{2}^{2}+\lambda_{2}^{2}\lambda_{3}^{2}+\lambda_{1}^{2}\lambda_{3}^{2}$ are first and second invariants, in which $\lambda_{1}$, $\lambda_{2}$, and $\lambda_{3}$ are stretching ratios in three orthotropic directions.

Model mesh and boundary conditions: For the crimping simulation, stents were meshed with 4-node linear tetrahedron elements (C3D4). A convergence study was performed on the DESolve stent to determine the mesh size by balancing computing cost and accuracy during crimping and degradation simulations. Considering four element sizes (i.e., 0.05 mm, 0.06 mm, 0.07 mm, and 0.08 mm) and examining the slopes of the initial linear-elastic stages of their force–diameter curves during the entire crimping process (see Figure S2 in Supplementary Materials), the 0.06 mm size was selected as the radial stiffness difference was 1.03% (i.e., 33.97 N/mm for the 0.05 mm size and 34.32 N/mm for 0.06 mm size), which was less than 5%. Consequently, the element numbers were 151,529, 172,758, 123,061, and 125,001 for the Absorb, DESolve, Igaki-Tamai, and Fantom stents, respectively. Radial displacement was applied on the sixteen rigid plates to compress the stent. One end of the stent was constrained to only allow radial deformation, while the other end was set free, and this also allowed the stent to displace in the axial direction of the stent. For the degradation simulation, the vessel and stent were meshed by brick elements (C3D8R), and the balloon was meshed by shell elements (S4R). The element numbers of the vessel and balloon were 13,050, with a size of 0.12 mm, and 1590, with a size of 0.12 mm, respectively. The element numbers were 9360, 10,512, 12,888, and 15,552, with a size of 0.05 mm for the Absorb, DESolve, Igaki-Tamai, and Fantom stents, respectively. Both vessel ends were constrained to reflect the in vivo environment. Six points in the middle supporting ring of the stents were constrained to only permit the stent’s radial expansion, while the two ends of the stent were set free. The contacts of the stent–plate, balloon–stent, and stent–vessel interfaces were defined as the penalty formulation, and a friction coefficient of 0.1 was used to simulate the tangential behavior of all contact pairs [35,36]. It is worth mentioning that the same contact type at the balloon–stent and stent–vessel interfaces was expected to not influence the degradation simulation as the balloon–stent interaction only existed during short-term stent expansion.

Crimping and degradation implementations: ABAQUS/Explicit was used to perform the crimping simulation and degradation simulation, of which the degradation model was coded via the VUMAT subroutine. The implementations are described as follows:

(1) Crimping simulation: The sixteen rigid plates were applied 0.8 mm displacement in the stent radial direction. To ensure deformation stability during the crimping process, the rigid plates were first slowly moved by 0.4 mm and remained stationary, and then were gradually advanced to a maximum of 0.8 mm.

(2) Degradation simulation: The simulation included two steps: one comprised stent expansion, and the other comprised stent degradation. A trapezoidal expansion history (black line in Figure 1g) was first imposed on the inner surface of the balloon to expand the PLA stent and the vessel. Then, human blood pressure (green line in Figure 1g) was fitted by referring to [37]:

(9) $$P(kPa)=3.5e^{-{\left(\frac{t(s)-0.1543}{0.1373}\right)}^{2}}+0.9784e^{-{\left(\frac{t(s)-0.4168}{0.07873}\right)}^{2}}+13.49e^{-{\left(\frac{t(s)-0.4487}{1.006}\right)}^{2}}$$

The fitted blood pressure was applied on the inner surfaces of the stent and vessel to simulate the main in vivo mechanical microenvironment, and stent degradation initiated. Since a minimum duration of 6 months was clinically required for the artery to recover under the pressure of the vessel and blood flow [4], the degradation period was thus set as 180 days. As mentioned in our previous work [25], the weak blood flow-induced shear effect on degradation was neglected. All input parameters in the simulations are listed in Table 2. Moreover, to quantify dynamic degradation, four degradation indices were adopted [25], namely the mean number average molecular weight, residual volume fraction, mean von Mises stress, and stent diameter.

## 3. Results and Discussions

### 3.1. Mechanical Behaviors of Stents in the Crimping Simulation

**Figure 2:**
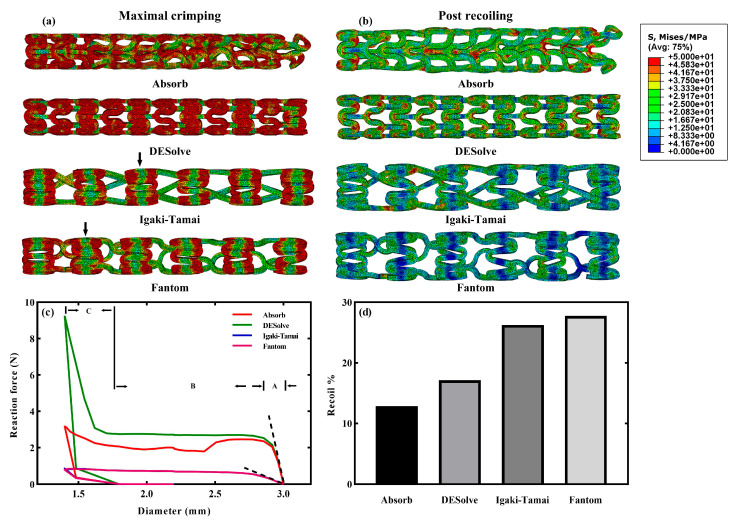
Crimping simulations of the four stents. The von Mises stresses of the stents at (**a**) the maximal crimping and (**b**) the post-recoiling states; (**c**) force–diameter curves of the stents during the entire crimping process; (**d**) recoiling rates after the crimping of stents. The following is noted in (**c**): stage (A) the linear elastic deformation is represented by the slope of the dashed line, which indicates the radial stiffness of the stent; stage (B) plastic deformation indicates the constant radial force of the stent; stage (C) plastic strengthening indicates an increase in the radial force of the stent to its peak as the minimal crimping diameter was gradually approached [30].

The von Mises stress distributions of the four stents at two states (the maximally compressed and post-recoiling) of the crimping simulation are shown in Figure 2a,b. In general, at both states, the Absorb and DESolve stents exhibited higher stress levels compared to the other two stents. In particular, the DESolve stent exhibited the highest stress level. Moreover, the high stress of the stent always occurred at the bend of the supporting rings (Figure 2a,b). At the maximally compressed state (Figure 2a), the stress distribution of the DESolve stent was different from that of the Absorb stent, even though they shared the same supporting ring. This indicated that the difference was attributed to the number of connecting struts when examining their structural features (Figure 1a vs. 1b and Figure S3 in Supplementary Materials). Moreover, the greater number of connecting struts in DESolve improved the stent’s radial stiffness, and the lower strut number in Absorb could not provide the structure with stability during crimping and further resulted in the irregular deformation of the supporting ring (Figure 2a). Interestingly, the greater supporting ring amplitude and lower supporting ring number of the Igaki-Tamai and Fantom stents resulted in regular patterns of the stress distribution; moreover, an apparent stress difference between the ring’s bend and the ring’s middle (indicated by the black arrows) was observed, and the connecting strut exhibited low stress. At the post-recoiling state (Figure 2b), all four stents radially recoiled together with a significant reduction in stress levels with respect to the maximal crimping state. Specifically, the Absorb stent remained irregular after recoiling due to the plastic deformation, and this again illustrated the structural instability of the Absorb stent during stenting.

The radial stiffness and recoiling rate after the crimping of stents were always evaluated because of the stent’s design. Here, the stiffnesses of the four stents were monitored by tracking the reaction force against the stent’s diameter during the entire crimping process (refer to Figure 2c). Compared to the other stents, the reaction force of the DESolve stent increased faster and reached the highest value of 9.2 N when it was maximally crimped. This was consistent with the highest von Mises stress observed in Figure 2a. The reaction force of the Absorb stent followed, but it declined during stent maintenance and ultimately reached 3.2 N. This phenomenon was attributed to the stent’s instability under compression due to the lower number of connecting struts. In contrast, the reaction forces of the Igaki-Tamai and Fantom stents shared an almost identical force–diameter curve, and they reached 0.9 N, respectively. Since the supporting rings of stents played a role in expanding vessels, the Absorb and DESolve stents sharing the same supporting ring exhibited identical radial stiffness, and this was also observed for the other two stents (see the marked slopes in Figure 2c). Moreover, the greatest force of the DESolve stent under the same diameter was a result of an increase in struts, and this is consistent with the results reported in [41]. Although greater stent thickness was reported to enhance the radial stiffness of the stent, the stent structures significantly affected the stiffness as well [42]. In particular, the 150 μm thickness of the Absorb and DESolve stents was less than the 170 μm thickness of the Igaki-Tamai and Fantom stents, but the former two exhibited greater stiffness than the latter two due to their different structures. Recoiling rates after the crimping of the four stents were calculated using |D_crimp_ − D_recoil_|/D_crimp_ × 100% (Figure 2d), where D_crimp_ and D_recoil_ are the stent diameters at the maximal crimping and post-recoiling states [30]. The post-recoiling diameters were 1.58 mm, 1.64 mm, 1.77 mm, and 1.79 mm for the Absorb, DESolve, Igaki-Tamai, and Fantom stents, respectively. Referring to the minimal crimping diameter of 1.4 mm, the recoiling rates were calculated as 12.86% for the Absorb stent, 17.14% for the DESolve stent, 26.26% for the Igaki-Tamai stent, and 27.77% for the Fantom stent. The similar rates of the Igaki-Tamai and Fantom stents were comparable to the reported 30% value of the PLA stents [43], and the higher recoiling rates were a result of the elastic deformation of larger unyielded PLA volumes in the Igaki-Tamai and Fantom stents compared to the other two stents (see Figure 2a,b). Regarding why these two stents had a larger volume of unyielded PLA, it is believed that their structural configurations still dominated, e.g., the greater ring amplitude and fewer rings and struts. In other words, the more compliant Igaki-Tamai and Fantom stents under crimping could result in more flexible deformation.

### 3.2. Degradation Evolutions of Stents in the Degradation Simulation

To quantitatively describe the degradation processes of the four stents, four degradation variables were calculated (referring to [25]) and collected: the mean normalized number average molecular weight $\bar{\beta }\left(t\right)$, the residual volume fraction of the stent $v_{r}(t)$, the mean von Mises stress of the stent $\bar{\sigma }\left(t\right)$, and the stent diameter $D\left(t\right)$. The degradation evolutions of the four indices are described in the following sections.

#### 3.2.1. Stress Distributions of Stents

**Figure 3:**
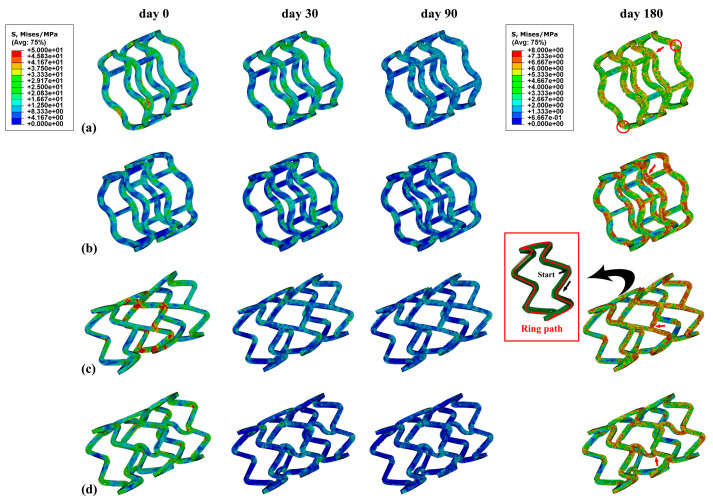
Von Mises stress distributions of the stents at four time points within 180 days: (**a**) Absorb, (**b**) DESolve, (**c**) Igaki-Tamai, and (**d**) Fantom stents.

The stress distributions of the stents at four time points (day 0, 30, 90, and 180) are shown in Figure 3. Generally, greater stress was observed at the bend of the supporting rings of all stents as a result of greater plastic deformation, and this was expected from and consistent with the reported literature [23,44]. Compared to the Absorb and DESolve stents, the plastic regions of the Igaki-Tamai and Fantom stents were larger at day 0, in particular, for the Igaki-Tamai stent. The reason was that their lower radial stiffnesses allowed them to radially expand more adequately under the identical balloon-expansion pressure, and thus caused the larger regions of plastic deformation. However, the general stress levels of the Igaki-Tamai and Fantom were lower compared to the other two stents as degradation proceeded. This was because of the initial faster degradation, which reduced the materials’ moduli and the stents’ diameters in the Igaki-Tamai and Fantom stents. In addition, decreasing the risk of fracture and fragment separation during degradation is very important [45,46]. Herein, the Absorb stent was considered the worst with respect to its degradation stability due to potential degradation-induced damage (circled in Figure 3).

**Figure 4:**
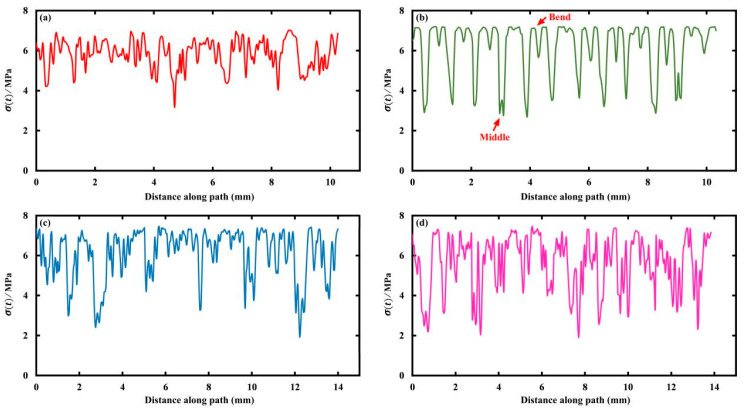
Von Mises stress along the ring paths of the middle ring at day 180: (**a**) Absorb, (**b**) DESolve, (**c**) Igaki-Tamai, and (**d**) Fantom stents.

In particular, the stress of the middle supporting rings along the ring path at day 180 is shown in Figure 4 for the four stents, and the starting point and direction of the path are indicated by the arrows in the inset of Figure 3. In detail, the stress of the Absorb stent fluctuated between 4 MPa and 7 MPa, while the other stents varied within a wider range. The reason was that, on the one hand, the Absorb stent was able to adequately expand with respect to the DESolve stent due to the lower number of connecting struts; on the other hand, the stress concentration more easily occurred in the Igaki-Tamai and Fantom stents due to the inclined and wavy connecting struts under stent expansion (refer to the 2D stent sheets in Figure S3 of Supplementary Materials). However, the DESolve stent exhibited more pronounced periodicity that corresponded to the ring’s configuration, and this indicated the degradation’s controllability. The periodicity of the DESolve stent resulted from the symmetrical 2D stent structure compared to the other three, and the apparent periodicity allowed us to control the degradation more easily (also refer to Figure S3 of Supplementary Materials). Moreover, the greatest stress of 7 MPa occurred at the ring’s bends, while the lowest stress of 3 MPa was observed in the middle of two adjacent bends.

#### 3.2.2. $\beta \left(t\right)$ Distributions of Stents

**Figure 5:**
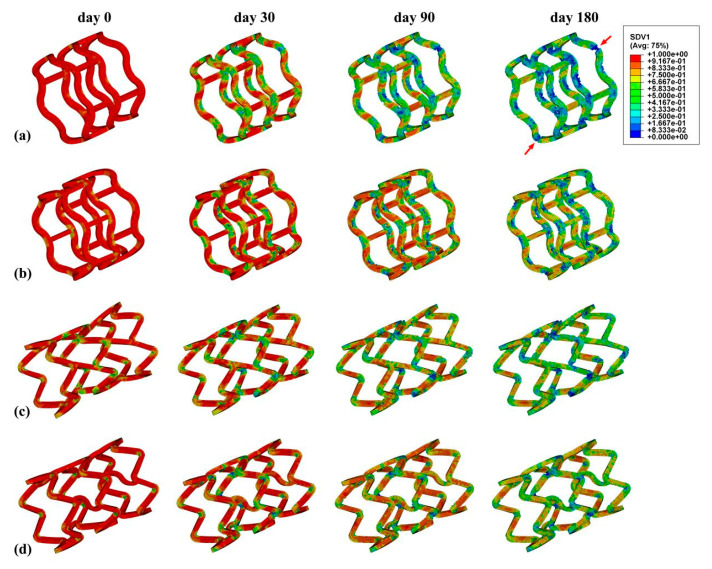
The $\beta \left(t\right)$ evolutions of the stent at four time points within 180 days: (**a**) Absorb, (**b**) DESolve, (**c**) Igaki-Tamai, and (**d**) Fantom stents.

To better understand the stent degradation, the $\beta \left(t\right)$ distributions of the stents at the four time points are shown in Figure 5. The elements at the supporting ring’s bend and the ring–strut joint degraded faster, and those at the connecting struts degraded slower; moreover, the Absorb stent failed more easily. This corresponded with the stress distribution in Figure 3 and further demonstrated that stress could accelerate the degradation of the stents.

**Figure 6:**
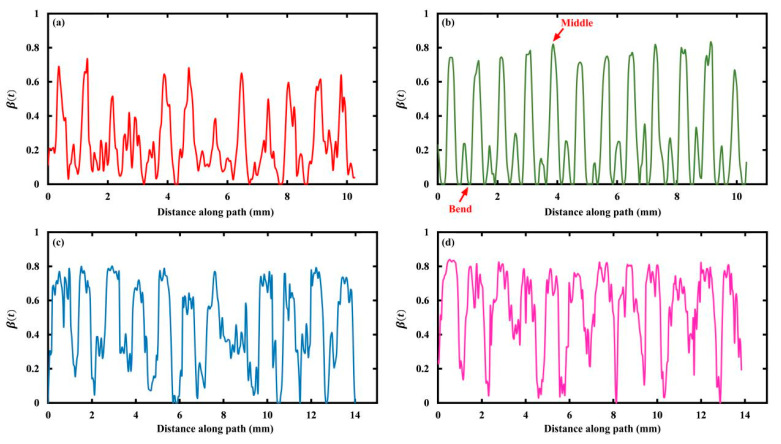
The $\beta \left(t\right)$ along the ring paths of the middle ring at day 180: (**a**) Absorb, (**b**) DESolve, (**c**) Igaki-Tamai, and (**d**) Fantom stents.

Similarly, Figure 6 shows $\beta \left(t\right)$ along the ring paths of the middle supporting rings of the four stents at day 180. Again, the periodicity of $\beta \left(t\right)$ is indicated due to the ring configurations, and a more remarkable periodicity is observed for the DESolve stent, highlighting the degradation’s controllability. However, the positions of the highest and lowest values of $\beta \left(t\right)$ were inverse to the stress counterparts in Figure 4. Namely, the highest value of 0.8 was located in the middle of two adjacent rings’ bends, and the lowest value of 0 was located at the bends. This was expected due to the negative relationship between stress and $\beta \left(t\right)$. In other words, higher stress resulted in faster degradation, which represented a lower $\;\beta \left(t\right)$ value. This is because the stress reduced the activation energy of PLA hydrolysis [28], and this is also expected when referring to Equation (2), where stress increased the degradation rate.

#### 3.2.3. Dynamic Evolutions of $\bar{\beta }(t)$

**Figure 7:**
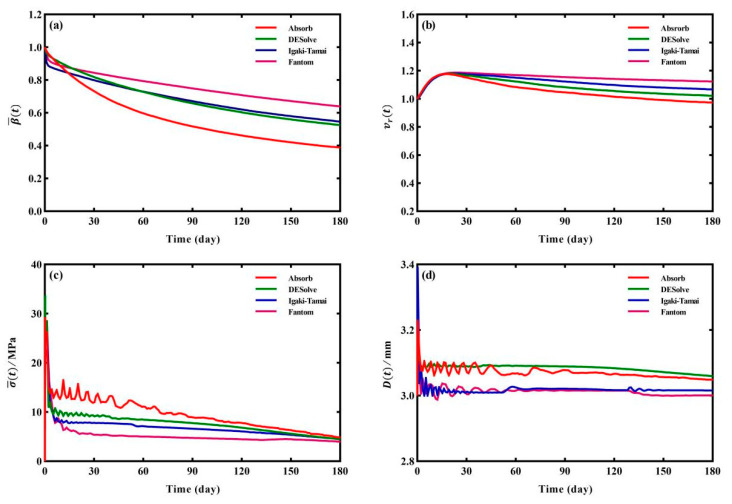
Evolutions of the indices of the four stents: (**a**) $\bar{\beta }\left(t\right)$, (**b**) $v_{r}(t)$, (**c**) $\bar{\sigma }\left(t\right)$, and (**d**) $D\left(t\right)$.

In order to quantitatively evaluate the degradation dynamics of the stents, the evolution curves of the four indices $\bar{\beta }(t)$, $v_{r}(t)$, $\bar{\sigma }\left(t\right)$, and $D\left(t\right)$ are shown in Figure 7. Generally, each of the four indices exhibited a similar tendency, which is consistent with experimental data [47]. At a very early stage (before day 5), the $\bar{\beta }(t)$ of the Igaki-Tamai and Fantom stents declined more sharply than the other two stents (Figure 7a), and this was attributed to the local higher stress induced by larger regions of plastic deformation (the stress at day 0 in Figure 3c,d). Subsequently, the $\bar{\beta }(t)$ of the two stents gradually became superior to the other two and finally decreased to 0.55 and 0.64 at day 180, respectively, while the $\bar{\beta }(t)$ of the Absorb and DESolve stents at day 180 were 0.39 and 0.52, respectively. The reason was that the initial faster degradation of the Igaki-Tamai and Fantom stents reduced the stent’s diameter, which resulted in lower general stress levels (Figure 3c,d); furthermore, the stress-induced degradation slowed the decrease in $\bar{\beta }(t)$. 

#### 3.2.4. Dynamic Evolutions of $v_{r}(t)$

Considering the swelling factor, $v_{r}(t)$ reached a maximum around 20 days and later gradually decreased (Figure 7b), which was roughly supported by the experimental observations in the literature [48], where the increase was instable within day 14. At day 180, $v_{r}(t)$ was 0.97, 1.02, 1.07, and 1.12 for the Absorb, DESolve, Igaki-Tamai, and Fantom stents, respectively. This was associated with the residual volume and attributed to the competition between the swelling-induced volume increase and degradation-induced volume decrease [25]. Interestingly, $v_{r}(t)$ varied more weakly compared to $\bar{\beta }(t)$. This was because stent elements were not removed until they satisfied the degradation criteria (i.e., Equation (5)), and their molecular weights continuously decreased, but the residual volumes of the stents were always increased thanks to the swelling effect. 

#### 3.2.5. Dynamic Evolutions of $\bar{\sigma }\left(t\right)$

The $\bar{\sigma }\left(t\right)$ of the stent is shown in Figure 7c. With the degradation progress, $\bar{\sigma }\left(t\right)$ decreased slowly to 4.85 MPa, 4.45 MPa, 4.41 MPa, and 3.97 MPa at day 180 for the Absorb, DESolve, Igaki-Tamai, and Fantom stents, respectively. Similarly to $\bar{\beta }(t)$, the lower general stress levels of the Igaki-Tamai and Fantom stents resulted from their initial faster degradation with respect to the other two stents (Figure 3). Actually, the initial faster degradation caused a decrease in the reduced material modulus (or radial stiffness), which could not maintain the vessel’s lumen during degradation, and this resulted in a smaller diameter. Thus, their general stress levels $\bar{\sigma }\left(t\right)$ were expected to be lower.

#### 3.2.6. Dynamic Evolutions of $D\left(t\right)$

The diameter $D\left(t\right)$ evolution of the four stents is plotted in Figure 7d. During the stent’s expansion, the diameters at peak expansion were 3.23 mm, 3.19 mm, 3.39 mm, and 3.38 mm for the Absorb, DESolve, Igaki-Tamai, and Fantom stents, respectively, and their counterparts at the post-recoiling state after expansion were 3.13 mm, 3.11 mm, 3.04 mm, and 3.07 mm. The recoiling rates after expansion were calculated as 3.02%, 2.47%, 10.38%, and 9.13% for the Absorb, DESolve, Igaki-Tamai, and Fantom stents, respectively. These percentages were comparable to the 4.19% reported in [30]. The expansion ratios were 1.04, 1.04, 1.01, and 1.02 times the initial lumen diameter of 3.00 mm. This was consistent with clinical operations, which required an expansion ratio between 1.0 and 1.1 [49]. At day 180, their diameters decreased to 3.05 mm, 3.06 mm, 3.02 mm, and 3.00 mm, respectively. This indicated that the DESolve stent exhibited superior lumen diameter maintenance ability compared to the other three stents, and the Igaki-Tamai and Fantom stents were close to the initial stent diameter of 3.0 mm, which represented a lower level of stress. Again, the smaller final diameters of the Igaki-Tamai and Fantom stents were attributed to their lower radial stiffness and the decreased material modulus. Noted is that the fluctuation of the mean stress and diameter were manifestations of the initial kinetic energy caused by the nature of the Abaqus/explicit solver [50].

#### 3.2.7. Von Mises Stress Distribution of Vessel

**Figure 8:**
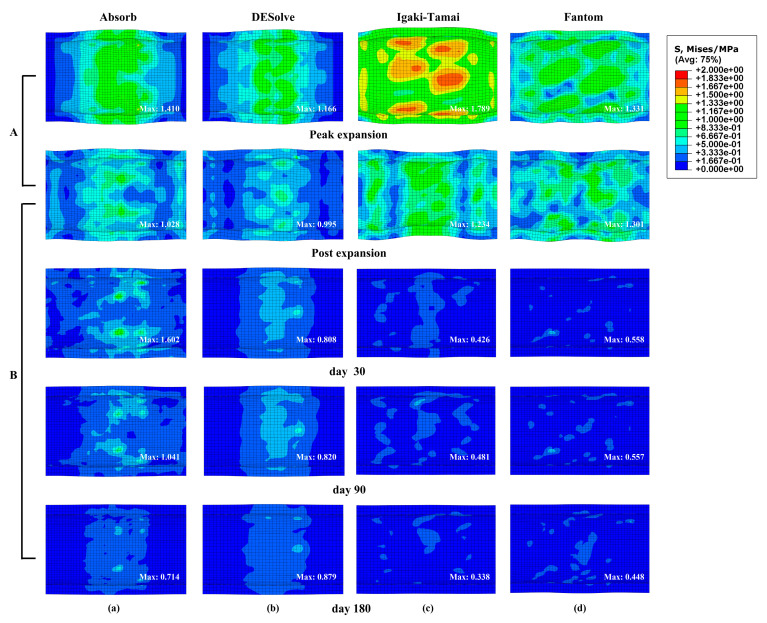
Von Mises stress distributions of the vessel at the expansion (panel **A**) and degradation (panel **B**) stages: (**a**) Absorb, (**b**) DESolve, (**c**) Igaki-Tamai, and (**d**) Fantom stents.

The von Mises stresses of the vessel during the balloon expansion (panel A) and degradation (panel B) stages are shown in Figure 8. The diameters at peak expansion were 3.23 mm, 3.19 mm, 3.39 mm, and 3.38 mm for the Absorb, DESolve, Igaki-Tamai, and Fantom stents, respectively. Due to the lower radial stiffness of the Igaki-Tamai and Fantom stents, their greater expansion resulted in the vessel’s higher stress distributions (panel A). After removing the balloon, under the coactions of the degradation and lower radial stiffness, their diameters reduced more greatly to 3.02 mm and 3.00 mm at day 180. This indicated their weaker diameter maintenance and the lower stress level of the vessel compared to the other two stents (panel B). This was consistent with the reported result in [51] that a larger ring amplitude and axial strut spacing induced lower stress levels on the vessel.

#### 3.2.8. Scoring Evaluations of Stents

**Figure 9:**
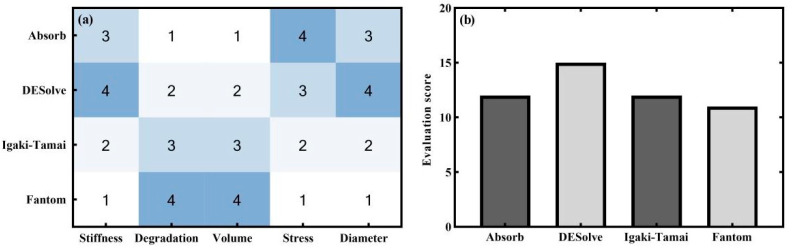
The indicator scores of the four stents. (**a**) Heat map and (**b**) evaluation score. Noted that the dark-to-light colors represented the score numbers from the best to the worst.

To evaluate the overall performance of the four stents, a pilot scoring system was proposed according to five indicators: radial stiffness, degradation, residual volume, mean stress level, and final diameter. The five indicators were common for evaluating the stents’ behaviors [46]. Greater radial stiffness, residual volume, mean stress level, and final diameter and slower degradation were considered to be beneficial in the sense of long-term stenting effects. Each indicator was ordered from the best to the worst by referring to Figure 2d and Figure 7, and they were correspondingly scored from 4 to 1. It is noted that the identical weights of the five indicators were qualitatively assigned due to their importance. All indicators of each stent are scored in Figure 9a, and the total scores of each stent are plotted in Figure 9b. It was readily observed that the DESolve stent achieved the highest score, and this indicated that the DESolve stent performed better among the four stents according to the current model and pilot scoring system. In addition, the degradation and residual volumes of the Absorb and DESolve were inferior to the other two stents, and this could also be a result of smaller thickness (150 μm vs. 170 μm), in addition to their greater mean stress levels. However, the effect of the thickness was not included as the commercial stents had a determinate thickness defined by the companies. 

## 4. Conclusions

In the present study, the performances of the crimping mechanical and degradation behaviors of four structured PLA BVS were comprehensively assessed by utilizing a custom finite element method. The results show that the stent’s design strongly affects its mechanical properties and degradation behaviors. Under the current framework, the DESolve stent performed better with respect to radial stiffness, diameter maintenance, and degradation controllability, even though the degradation and reduction of residual volume were faster compared to the Igaki-Tamai and Fantom stents. The findings provide a theoretical method for studying the mechanical and degradation behaviors of PLA BVS, and could be potentially useful in advancing BVS design and clinical protocols.

Compared to the existing studies, the current study developed relative to two aspects: (1) This model explicitly included physio-chemical factors with respect to the continuum damage mechanics approach [52] and integrated the factors into the degradation rate compared to the pseudo first-order kinetics, which only consider a constant degradation rate [53]. (2) The results obtained from the current model were able to provide insights to understand the entire dynamic degradation process, and a pilot scoring system could be helpful for selecting a suitable commercial stent despite the qualitative weight contributions of the five important indicators. Indeed, there were limitations: (1) The study was numerical, and an in vivo stent degradation experiment should be performed. (2) More factors potentially influencing PLA degradation should be included, such as environmental pH value and layered vessels. (3) The weights of the five indicators in the pilot scoring system should be quantified. Despite these limitations, the current findings were still useful in understanding the complex degradation behaviors of PLA BVS, which was very difficult to characterize via in vivo experiments.

## Figures and Tables

**Table 1:** Geometrical parameters of the four stents [22,29].

Stent Type	Stent Length (mm)	Strut Width (μm)	Strut Thickness (μm)	Number of Rings	Number of Connecting Struts
Absorb	10.5	200	150	11	3
DESolve	10.5	200	150	11	6
Igaki-Tamai	10	200	170	6	3
Fantom	10	200	170	6	3

**Table 2:** Input parameters of the degradation analysis.

Input Parameters	Parameters	Values	Unit
Elastic constants of balloon	C10 C01	1.069 [33] 0.711 [33]	MPa
Elastic constants of blood vessel	C10 C01	1.023 [25] 0.710 [25]	MPa
Young’s modulus of stent	*E*_s_(0)	3.0 [38]	GPa
Young’s modulus of solution	$E_{water}$	10 [25]	MPa
Poisson’s ratio of stent	$v_{s}$	0.3 [25]	-
Poisson’s ratio of solution	$v_{water}$	0.49 [25]	-
Density of blood vessel	$\rho_{vessel}$	1.066 [25]	g·cm^−3^
Density of balloon	$\rho_{balloon}$	1.07 [34]	g·cm^−3^
Density of dry PLA	$\rho_{dry}$	1.2 [31]	g·cm^−3^
Density of solution or water	$\rho_{water}$	1.0 [25]	g·cm^−3^
Degradation rate constant	$\lambda_{0}$	0.003 [39]	day^-1^
Size effect coefficient	*k*	0.12 *	-
Degradation threshold	$\beta_{thre}$	0.01 [25]	-
Initial diffusion coefficient	$\varphi_{0}$	1.2 × 10^−9^ [40]	m^2^·day^-1^
Initial yield strength of stent	$\sigma_{y}(0)$	50 [31]	MPa
Material constant diffusivity	θ	9.43 [28]	-
Proportional coefficient	ϕ	1.225 [32]	-
Fatigue-induced decrease coefficient of the strength	*η*	0.99 *	

* Estimated.

## Data Availability

The data that support the findings of this study are available within the article.

## Supplementary Materials

The following supporting information can be downloaded at: https://www.mdpi.com/article/10.3390/jfb15050135/s1, Figure S1: The current strength decay curve of PLA; Figure S2: Mesh density analysis of the DESolve stent with four element sizes. (a) The diameter-force curves of the DESolve stent during the whole crimping process, (b) Stress distribution patterns; Figure S3: The planar expansion of the four stents. Noted that the structure of the DESolve stent was strictly symmetric, and it enabled the apparent periodic degradation behaviors.
