# Supplementary material for: In Silico Evaluation of In Vivo Degradation Kinetics of Poly(Lactic Acid) Vascular Stent Devices
Source: J Funct Biomater. 2024 May 17;15(5):135. doi: 10.3390/jfb15050135 (PMC11122488; doi:10.3390/jfb15050135)
Supplement: Supplementary file 1 [file jfb-15-00135-s001.zip › jfb-2962479-supplementary.pdf]

In Silico Evaluation of In Vivo Degradation Kinetics of Poly(Lactic Acid) Vascular Stent Devices

Shicheng He [1], Lingling Wei [2], Guixue Wang [3], Nicola M. Pugno [4,5], Qiang Chen [1,*] and Zhiyong Li [1,6,7,*]

1 Biomechanics Laboratory, School of Biological Science and Medical Engineering, Southeast University, Nanjing 210096, China
2 School of Food and Biological Engineering, Hefei University of Technology, Hefei 230601, China
3 Key Laboratory for Biorheological Science and Technology of Ministry of Education, State and Local Joint Engineering Laboratory for Vascular Implants, Bioengineering College of Chongqing University, Chongqing 400030, China
4 Laboratory for Bioinspired, Bionic, Nano, Meta Materials and Mechanics, University of Trento, Via Mesiano 77, 38123 Trento, Italy
5 School of Engineering and Materials Science, Queen Mary University of London, Mile End Road, London E1 4NS, UK
6 School of Mechanical, Medical and Process Engineering, Queensland University of Technology, Brisbane, QLD 4001, Australia
7 Faculty of Sports Science, Ningbo University, Ningbo 315211, China
* Correspondence: cq@seu.edu.cn (Q.C.); zhiyong.li@qut.edu.au (Z.L.)

Supplementary Materials

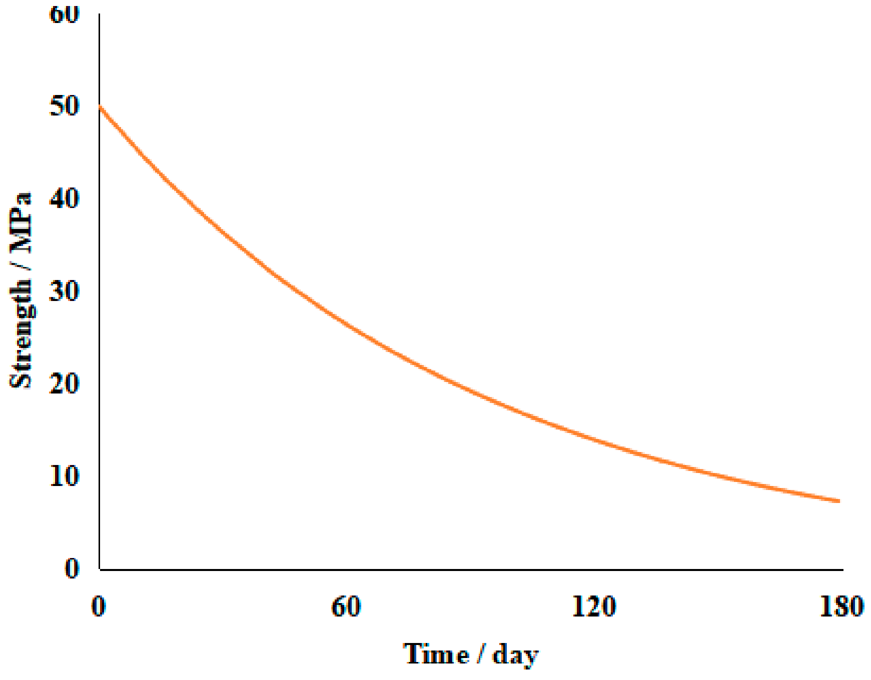

Figure S1 The current strength decay curve of PLA

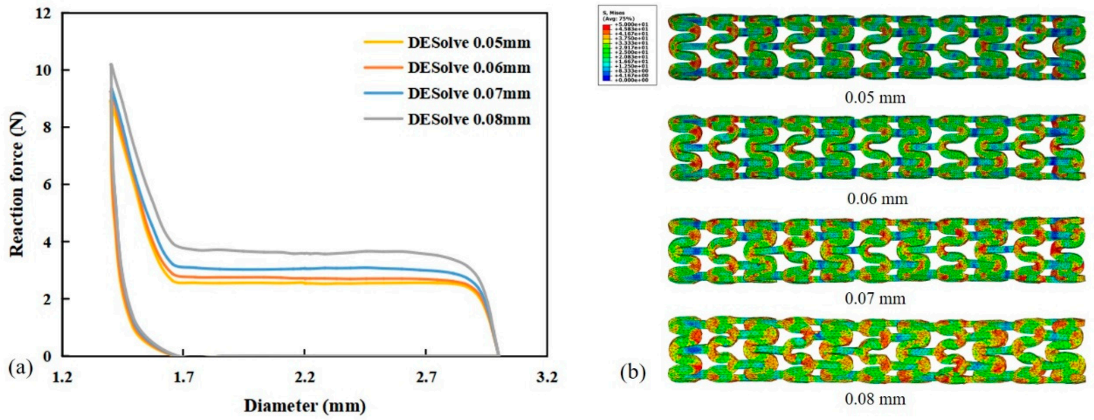

Figure S2. Mesh density analysis of the DESolve stent with four element sizes. (a) The diameter-force curves of the DESolve stent during the whole crimping process, (b) Stress distribution patterns.

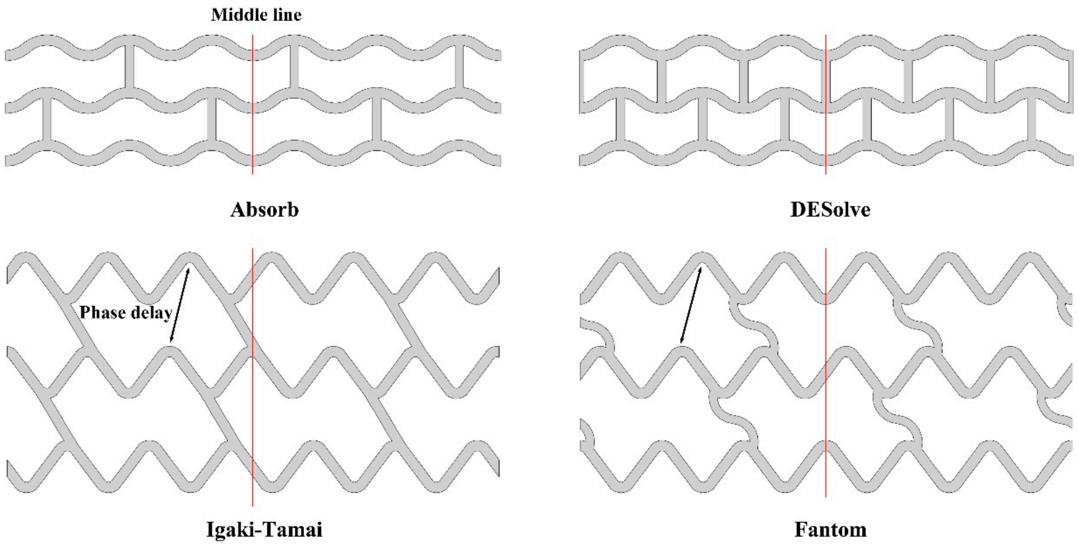

Figure S3. The planar expansion of the four stents. Noted that the structure of the DESolve stent was strictly symmetric, and it enabled the apparent periodic degradation behaviors.
